# The Wasting Diseases of Infants and Children

**Published:** 1885-08

**Authors:** 


The Wasting Diseases of Infants and Children. By Eustace Smith, M. D., London. Fourth Edition. New York: William Wood & Co., 56 and 58 Lafayette Place. 1885.

This book of 278 pages, replete with useful practical lessons respecting the management of children, both as to their regimen and medical treatment, has stood the test of close examination by the profession, and in this fourth edition the text has been revised, and many alterations and additions have been introduced.

When it is considered how large a place infantile disorders have in the mortuary statistics of this country, the importance of a work, such as this, for arresting the fatal tendencies of disease at this early period of life should be duly appreciated.

The class of troubles included in this treatise, consisting of hereditary and acquired affections of a constitutional character, needs to be studied thoroughly by the physician, and Messrs. William Wood & Co. have made a most useful addition to the resources of the practitioner by placing this work at his side for combatting the maladies of early life.

